# Spatial Clusters of High Prevalences of Overweight and Obesity Among Children in Indonesia

**DOI:** 10.7759/cureus.57370

**Published:** 2024-04-01

**Authors:** Sofi Oktaviani, Mayumi Mizutani, Ritsuko Nishide, Susumu Tanimura

**Affiliations:** 1 Department of Public Health Nursing, Graduate School of Medicine, Mie University, Tsu, JPN; 2 Department of Nursing, Indramayu College of Health Sciences, Indramayu, IDN; 3 Department of Integrated Health Sciences, Graduate School of Medicine, Nagoya University, Nagoya, JPN; 4 Department of Nursing, Nagoya University of Arts and Sciences, Nagoya, JPN

**Keywords:** spatial clusters, overweight, obesity, indonesia, child

## Abstract

Objective

Childhood obesity has emerged as a pressing health concern in both high-income and lower-middle-income countries, including Indonesia. The prevalence of overweight and obesity among school children aged 5-12 years has been increasing in Indonesia, with 20% of Indonesian children identified as obese or overweight in 2018. Therefore, addressing this problem will be challenging. This study aims to identify district- and city-level clusters with high prevalences of overweight and obese children aged 5-12 years in Indonesia.

Methodology

This is an ecological study that utilizes secondary data from the 2018 Basic Health Research report conducted by the Indonesian Ministry of Health. We included 514 districts and cities to detect district- and city-level clusters. Spatial cluster analysis was performed using restricted flexible scan statistics to identify clusters with high prevalences of childhood overweight and obesity in Indonesian districts and cities.

Results

The findings reveal that childhood overweight and obesity are not randomly distributed. The study detected 20 clusters with high prevalences of childhood overweight and 36 clusters of obesity, with a particular concentration in Western Indonesia. A primary cluster of childhood overweight occurred in Sijunjung, Tanah Datar, Agam, Pasaman, South Solok, Dharmasraya, West Pasaman, Sawah Lunto City, Padang Panjang City, and Kampar. A primary cluster of obesity occurred in Mandailing Natal, South Tapanuli, Central Tapanuli, North Tapanuli, Labuhan Batu, North Padang Lawas, Padang Lawas, North Labuhan Batu, West Pasaman, and Rokan Hilir.

Conclusions

This study found 20 clusters with high prevalences of childhood overweight and 36 clusters of obesity in Indonesia. Implementing health promotion programs in the identified cluster regions will be crucial to effectively addressing the growing problem of childhood obesity in Indonesia.

## Introduction

During the 2000s, a noticeable increase was observed in the prevalence of overweight and obesity among children and adolescents, especially in middle-income countries, with the most significant increases found in East, South, and Southeast Asia [1]. In Indonesia, the classifications of *overweight* and *obesity* were based on the criteria outlined by the Indonesian government, aligning with the World Health Organization’s standards [2]. In 2018, 20% of Indonesian children aged 5-12 years were identified as either overweight or obese [3]. The COVID-19 pandemic has only exacerbated these trends, as community lockdowns have led to increased consumption of unhealthy foods and a reduction in children’s physical activities [4,5]. Meanwhile, evidence is accumulating that childhood obesity profoundly affects the development of adverse health outcomes. For example, obese children are 3.89 times more likely to develop diabetes in adulthood [6] and 5.21 times more likely to be obese in adulthood [7], and childhood extreme obesity will increase the risk of pediatric hypertension threefold [8].

A systematic review study [9] identified that overweight and obesity among children results from a complex interplay between pre- and post-natal factors (e.g., high maternal weight gain during pregnancy and high birth weight), personal behaviors (e.g., excessive intake of carbohydrates, insufficient physical activity, prolonged screen time, and inadequate sleep duration), psychosocial factors (e.g., parental misperception of their child’s body weight), and sociodemographic status (e.g., low social status). Recently, several studies have employed geographical data to gain a deeper understanding of the determinants and patterns of childhood obesity [10-13]. These studies indicate that the prevalence of childhood overweight and obesity showed a clustering pattern and that implementing targeted interventions in specific cluster regions could be an effective method for addressing childhood overweight and obesity.

However, there are several limitations in the existing literature that must be addressed. First, most previous studies have been conducted at the provincial level (subnational administrative level). Conducting comprehensive spatial cluster analyses that encompass all districts and cities within a country is essential. This approach will allow for more accurate measurements of the nature and intensity of geographical interconnections among data across a broader geographical area. Future studies should aim to provide further clarification through spatial analysis on the national level. Second, although child obesity (9.2%) [3] is an increasingly visible issue in Indonesia, there is a lack of comprehensive research on this topic, including geographical assessments. Therefore, this study aims to identify district- and city-level clusters with high prevalences of overweight and obese children aged 5-12 years in Indonesia.

## Materials and methods

Study design and data sources

This is an ecological study that utilizes secondary data obtained from 514 districts and cities that were recorded for the 2018 Basic Health Research report authored by the Indonesian Ministry of Health [3]. In this survey, 300,000 responses from households, excluding those belonging to specific census blocks and categories (e.g., orphanages, dormitories, and hostels) [14], were gathered using a two-stage sampling process. During the first stage, 180,000 (25%) members from a census block of 720,000 (2010 census data) were selected by applying probability proportional to size. Subsequently, the survey selected 30,000 census blocks from both urban and rural areas by applying proportionality to size. In the second stage of the survey, ten households from the target population of each census block were selected for participation through systematic sampling. To maintain the representativeness of household characteristics and ensure diversity among the selected households, the team employed an implicit stratification method, selecting ten households based on the highest education levels completed by the heads of household [3].

The survey also measured the prevalence of overweight and obesity among children aged 5-12 years in 514 districts and cities across Indonesia. The Indonesian government classified the nutritional status of each child using the World Health Organization’s criteria for overweight (1 SD < BMI-for-age z-score [BAZ] ≤ 2 SD) and obese (BAZ > 2 SD) children [2,3]. The boundaries and geographic data collected in this study were from the official Humanitarian Data Exchange - an open-data platform that offers administrative information for all countries, including data gathered at various subdivision levels [15].

Data processing

We added district and city IDs from Indonesia’s Central Bureau of Statistics to the prevalence data on overweight and obesity among children. The map data included polygons of the geographical borders of districts and cities, which were given unique ID numbers. Then, using these IDs for 514 districts and cities, we merged the data on the prevalence of overweight and obesity with map data.

Spatial cluster detection

The spatial scan statistics method presented by Kulldorff represents a widely employed and powerful method for detecting clusters [16]. The traditional spatial scan statistics method, however, considers only circular clusters. However, most geographic areas, including those in Indonesia, are irregularly shaped. Therefore, this study applied a flexible scan statistics method to detect arbitrarily shaped clusters in Indonesia. The restricted flexible scan statistics method proposed by Tango and Takahashi can detect cluster areas using flexible shapes [17]. By employing these methods, adjacent districts and cities could be connected to create a flexibly shaped window for the centroid of each district and city. For each district and city in Indonesia, a set of irregularly shaped windows was formed, with the maximum number of possible regions to be included in a potential cluster set at 20 (*k* = 20). We developed a neighbor matrix for districts and cities from a list of polygons that shared a side with another adjacent polygon. The *P*-value of restricted scan statistics was calculated using Monte Carlo hypothesis testing with 999 replications. This study set a significant cluster at a *P*-value < 0.05. We identified cluster regions in Indonesia using the R *smerc* package [18]. All data processing, analyses, and visualizations were performed using R version 4.3.0 [19].

Ethical considerations

The data used in this study were publicly available and did not contain any information that could be used to identify individuals. Therefore, ethical clearance was not required for this study.

## Results

**Figure 1:**
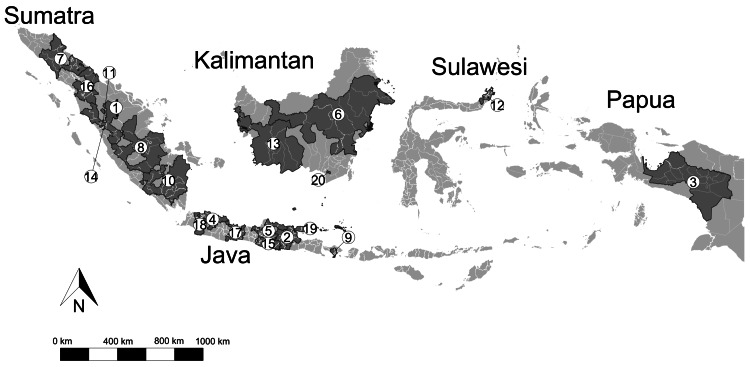
Spatial clusters of high childhood overweight prevalences at district and city levels in Indonesia. *Note*: The dark gray color represents district- and city-level clusters of high overweight prevalences among children aged 5-12 years. A number was assigned to the cluster area for identification. Image credit: All authors.

**Figure 2:**
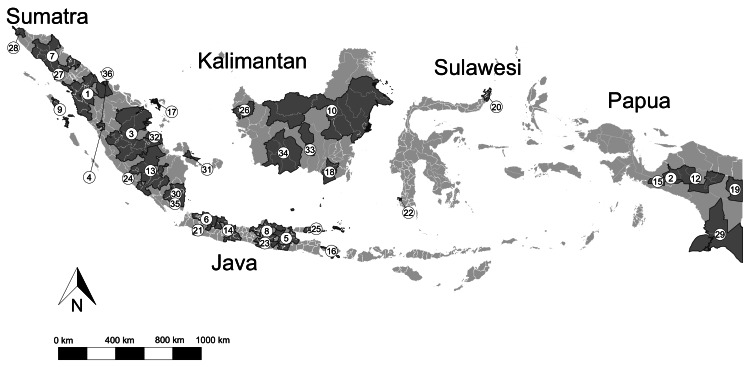
Spatial clusters of high childhood obesity prevalences at district and city levels in Indonesia. *Note*: The dark gray color represents district- and city-level clusters of high obesity prevalences among children aged 5-12 years. A number was assigned to the cluster area for identification. Image credit: All authors.

The mean overweight prevalence among the 514 districts and cities was 10.3% (SD = 4.2), while the mean obesity prevalence was 8.6% (SD = 5.5). The districts with the lowest and highest prevalences of overweight were identified as Dompu (0.9%) and Tolikara (33.5%) districts, respectively. Meanwhile, the districts with the lowest and highest prevalences of obesity were Central Sumba and Buton (0%), and Paniai (73.9%) districts, respectively. This study clarified that the prevalence of overweight and obesity among children aged 5-12 years in Indonesia was clustered. These clusters are represented with a dark gray color in Figures 1-2. Using flex scan statistics, we identified 20 overweight clusters and 36 obesity clusters (Tables 1-2, respectively).

**Table 1:** Spatial clusters of high childhood overweight prevalences at district and city levels in Indonesia. RR, relative risk; LLR, log-likelihood ratio

Clusters	Province (District/City)	Population	Cases	RR	LLR	*P*-value
1	West Sumatra (Sijunjung, Tanah Datar, Agam, Pasaman, South Solok, Dharmasraya, West Pasaman, Sawah Lunto City, and Padang Panjang City) and Riau (Kampar)	282,864	28,291.9	1.4	816.7	0.001
2	East Java (Tulungagung, Blitar, Kediri, Pasuruan, Sidoarjo, Mojokerto, Jombang, Nganjuk, Madiun, Bojonegoro, Lamongan, Gresik, Kediri City, Pasuruan City, Surabaya City, and Batu City)	6,074	934.7	1.9	165.2	0.001
3	Papua (Jayawijaya, Nabire, Paniai, Puncak Jaya, Asmat, Yahukimo, Tolikara, Waropen, Nduga, Lanny Jaya, Central Mamberamo, Yalimo, Puncak, Dogiyai, Intan Jaya, and Deiyai)	2,721	520.9	2.3	159.2	0.001
4	Jakarta (South Jakarta, East Jakarta, Central Jakarta, West Jakarta, and North Jakarta), West Java (Indramayu, Subang, Purwakarta, Karawang, Bekasi, West Bandung, Bandung City, Bekasi City, Depok City, and Cimahi City), and Banten (South Tangerang City)	6,956	988.3	1.7	135.3	0.001
5	Central Java (Boyolali, Sukoharjo, Karanganyar, Sragen, Grobogan, Rembang, Pati, Kudus, Semarang, Kendal, Surakarta City, Salatiga City, and Semarang City) and East Java (Magetan, Ngawi, and Tuban)	4,960	670.9	1.6	76.6	0.001
6	West Kalimantan (Sintang), Central Kalimantan (North Barito, Gunung Mas, East Barito, and Murung Raya), South Kalimantan (Tabalong), and East Kalimantan (Paser, West Kutai, Kutai Kartanegara, East Kutai, Berau, Mahakam Hulu, and Balikpapan City)	3,550	515.9	1.8	75.8	0.001
7	Aceh (South Aceh, Southeast Aceh, East Aceh, Southwest Aceh, Gayo Lues, Aceh Tamiang, and Langsa City) and North Sumatra (Simalungun, Karo, Deli Serdang, Serdang Bedagai, Medan City, and Binjai City)	7,653	907.9	1.4	57.6	0.001
8	Riau (Indragiri Hulu), Jambi (Kerinci, Merangin, Batang Hari, Muaro Jambi, East Tanjung Jabung, Tebo, and Jambi City), South Sumatra (Musi Rawas, Musi Banyuasin, and Lubuklinggau City), and Bengkulu (Lebong)	3,531	468.6	1.6	49.5	0.001
9	Bali (Tabanan, Badung, Gianyar, and Denpasar City)	1,564	243.6	1.9	44.3	0.001
10	South Sumatra (Ogan Komering Ilir, Muara Enim, Lahat, South Ogan Komering Ulu, East Ogan Komering Ulu, and Prabumulih City), Bengkulu (Kaur), and Lampung (Central Lampung, North Lampung, Way Kanan, Tulangbawang, Pringsewu, Mesuji, West Tulang Bawang, and Metro City)	4,427	537.9	1.5	38.5	0.001
11	West Sumatra (Payahkumbuh City)	15,089	1,540.6	1.2	34.6	0.001
12	North Sulawesi (Minahasa, South Minahasa, North Minahasa, Manado City, Bitung City, and Tomohon City)	2,122	290.4	1.7	34.5	0.001
13	West Kalimantan (Mempawah, Ketapang, Melawi, Kubu Raya, and Pontianak City) and Central Kalimantan (West Kotawaringin, East Kotawaringin, Sukamara, Lamandau, and Seruyan)	3,475	425.0	1.5	31.3	0.001
14	West Sumatra (Solok City)	8,920	922.3	1.2	23.1	0.001
15	Central Java (Klaten and Wonogiri), Yogyakarta (Bantul, Gunung Kidul, and Sleman), and East Java (Pacitan, Ponorogo, and Trenggalek)	2,387	289.6	1.5	20.5	0.001
16	North Sumatra (South Tapanuli, North Tapanuli, Labuhan Batu, Asahan, North Padang Lawas, Padang Lawas, North Labuhan Batu, Tanjung balai City, and Padangsidimpuan City)	3,062	354.6	1.4	19.6	0.001
17	West Java (Cirebon, Pangandaran, and Banjar City) and Central Java (Cilacap, Banyumas, Purbalingga, Pemalang, and Brebes)	3,497	391.1	1.3	17.5	0.001
18	West Java (Bogor, Sukabumi, Bogor City, and Sukabumi City) and Banten (Tangerang)	2,946	331.7	1.4	15.5	0.005
19	East Java (Bangkalan, Sampang, Pamekasan, and Sumenep)	1,421	176.7	1.5	14.0	0.014
20	South Kalimantan (Banjar Baru City)	226	43.2	2.3	13.1	0.024

**Table 2:** Spatial clusters of high childhood obesity prevalences at district and city levels in Indonesia. RR, relative risk; LLR, log-likelihood ratio

Clusters	Province (Districts/Cities)	Population	Cases	RR	LLR	*P*-value
1	North Sumatra (Mandailing Natal, South Tapanuli, Central Tapanuli, North Tapanuli, Labuhan Batu, North Padang Lawas, Padang Lawas, and North Labuhan Batu), West Sumatra (West Pasaman), and Riau (Rokan Hilir)	61,239	5,764.5	1.5	431.3	0.001
2	Papua (Paniai)	268	198.2	11.5	394.4	0.001
3	West Sumatra (South Solok and Dharmasraya), Riau (Indragiri Hulu, Indragiri Hilir, and Pelalawan), and Jambi (Merangin, Sarolangun, Batang Hari, Muaro Jambi, Tebo, Bungo, and Jambi City)	52,319	4,736.8	1.4	284.1	0.001
4	West Sumatra (Solok City)	8,920	1,123.9	1.9	225.7	0.001
5	East Java (Tulungagung, Blitar, Pasuruan, Sidoarjo, Mojokerto, Jombang, Nganjuk, Bojonegoro, Gresik, Blitar City, Pasuruan City, Mojokerto City, Surabaya City, and Batu City)	5,003	717.9	2.2	197.7	0.001
6	Jakarta (South Jakarta City, East Jakarta City, Central Jakarta City, West Jakarta City, and North Jakarta City), West Java (Subang, Karawang, Bekasi, West Bandung, Bekasi City, and Depok City), and Banten (Tangerang, Tangerang City, and South Tangerang City)	7,378	937.7	2.0	192.3	0.001
7	Aceh (South Aceh, Southeast Aceh, East Aceh, Central Aceh, Southwest Aceh, Gayo Lues, Nagan Raya, and Langsa City) and North Sumatra (Karo, Deli Serdang, Serdang Bedagai, Medan City, and Binjai City)	6,986	755.8	1.7	93.9	0.001
8	Central Java (Sukoharja, Karanganyar, Sragen, Grobogan, Rembang, Pati, Kudus, Demak, Semarang, Kendal, Surakarta City, Salatiga City, and Semarang City) and East Java (Magetan, Ngawi, and Tuban)	5,065	584.9	1.8	90.4	0.001
9	North Sumatra (Nias, South Nias)	544	131.6	3.8	87.3	0.001
10	West Kalimantan (Sintang and Kapuas Hulu), Central Kalimantan (North Barito and Gunung Mas), and East Kalimantan (West Kutai, Kutai Kartanegara, East Kutai, Berau, North Penajam Paser, Mahakam Ulu, Balikpapan City, Samarinda City, and Bontang City)	3,941	472.2	1.9	81.5	0.001
11	West Sumatra (Tanah Datar, and Sawah Lunto City)	46,257	3641.8	1.2	77.7	0.001
12	Papua (Jayawijaya, Puncak Jaya, Nduga, Central Mamberamo, Yalimo, Puncak, and Intan Jaya)	1,013	177.2	2.7	71.9	0.001
13	South Sumatra (Ogan Komering Ulu, Muara Enim, Lahat, Musi Rawas, Musi Banyuasin, East Ogan Komering Ulu, Palembang City, Prabumulih City, and Lubuklinggau City) and Bengkulu (South Bengkulu, Rejang Lebong, and Kaur)	3,978	451.9	1.8	66.1	0.001
14	West Java (Cirebon, Sumedang, Indramayu, Pangandaran, Cirebon City) and Central Java (Cilacap, Banyumas, and Brebes)	3,251	371.2	1.8	55.1	0.001
15	Papua (Dogiyai)	192	54.5	4.4	43.9	0.001
16	Bali (Tabanan, Badung, Gianyar, Klungkung, Buleleng, and Denpasar City)	2,142	254.2	1.8	42.5	0.001
17	Kepulauan Riau (Batam City)	1,207	165.9	2.1	41.1	0.001
18	South Kalimantan (Tanah Laut, Banjar, Tapin, Banjarmasin City, and Banjar Baru City)	1,768	207.9	1.8	33.8	0.001
19	Papua (Pegunungan Bintang)	126	38.3	4.7	33.5	0.001
20	North Sulawesi (Minahasa, North Minahasa, Manado City, and Tomohon City)	1,473	179.8	1.9	32.6	0.001
21	West Java (Sukabumi)	616	94.0	2.4	29.4	0.001
22	South Sulawesi (Makassar City)	1,457	172.2	1.8	28.4	0.001
23	Central Java (Klaten, Wonogiri), Yogyakarta (Gunung Kidul, Sleman, and Yogyakarta City) and East Java (Ponorogo)	1,782	200.3	1.7	28.2	0.001
24	Bengkulu (Bengkulu City)	521	82.5	2.5	27.7	0.001
25	East Java (Pamekasan, Sumenep)	633	91.1	2.2	25.1	0.001
26	West Kalimantan (Landak, Mempawah, and Pontianak City)	1,104	133.0	1.9	23.2	0.001
27	Aceh (Subulussalam City)	134	31.7	3.7	20.5	0.001
28	Aceh (Aceh Besar and Banda Aceh)	773	97.7	1.9	19.4	0.001
29	Papua (Merauke and Mappi)	503	69.1	2.1	17.1	0.001
30	Lampung (East Lampung, Central Lampung, North Lampung, Tulangbawang, West Tulang Bawang, and Metro City)	2,243	215.6	1.5	16.4	0.001
31	Kepulauan Bangka Belitung (Central Bangka and Pangkal Pinang City)	550	69.9	2.0	14.1	0.007
32	Jambi (East Tanjung Jabung)	178	32.3	2.8	13.9	0.009
33	Central Kalimantan (Palangka Raya City)	368	51.2	2.2	13.0	0.017
34	Central Kalimantan (West Kotawaringin, East Kotawaringin, Sukamara, Lamandau, and Seruyan)	1,493	147.9	1.5	12.8	0.018
35	Lampung (Bandar Lampung City)	570	69.3	1.9	12.4	0.023
36	West Sumatra (Payakumbuh City)	15,089	1,121.1	1.2	11.7	0.037

As seen in Figure 1 and Figure 2, most of the clusters were located on Sumatra Island and Java Island, as well as in West Kalimantan and Middle Kalimantan, in Western Indonesia. East Java Province displayed the highest number of districts and cities with overweight clusters (*n* = 26), followed by Central Java (*n* = 20), and West Java (*n* = 17). In the case of obesity, the highest number of clusters was recorded in East Java (*n* = 20), Central Java (*n* = 17), and North Sumatra (*n* = 15).

The primary overweight cluster included Sijunjung, Tanah Datar, Agam, Pasaman, South Solok, Dharmasraya, West Pasaman, Sawah Lunto City, Padang Panjang City, and Kampar, which recorded a relative risk (RR) of 1.4 and a log-likelihood ratio (LLR) of 816.7 at a *P*-value of 0.001 (Table 1). Meanwhile, the primary obesity cluster included Mandailing Natal, South Tapanuli, Central Tapanuli, North Tapanuli, Labuhan Batu, North Padang Lawas, Padang Lawas, North Labuhan Batu, West Pasaman, and Rokan Hilir, with an RR of 1.5 and an LLR of 431.3 at a *P*-value of 0.001.

## Discussion

To the best of our knowledge, this study represents the first effort to apply geographical analysis to identifying obesity and overweight clusters among children in Indonesia. The results of the restricted flexible scan statistics found that the prevalences of overweight and obesity were clustered in specific districts and cities. We detected 20 cluster areas for childhood overweight and 36 cluster areas for obesity, most of which were concentrated in Western Indonesia.

We now offer several potential explanations for the distribution of clusters. First, the disparity of socioeconomic status between Western Indonesia and Middle/Eastern Indonesia might be responsible for cluster distributions within the country. Meanwhile, a study conducted in a middle-income country found that socioeconomic status can contribute to geographic disparities in the prevalence of obesity among children [10]. Regarding socioeconomic status, Western Indonesia, which encompasses Sumatra, Java, and Bali, is generally more affluent and prosperous compared to the eastern parts of Indonesia. A cross-sectional study determined that the human development index in Western Indonesia is 71.87, while it is only 67.63 in Eastern Indonesia. Furthermore, the poverty rate in Western Indonesia is lower than in Eastern Indonesia-9.27% and 12.63%, respectively [20]. Consequently, socioeconomic inequality within Indonesia emerges as one of the potential causes of overweight and obesity clusters in Western Indonesia. Second, it is important to consider the rapid urbanization that is occurring predominantly on the island of Java, which is part of Western Indonesia [21]. Several studies, including systematic reviews, have indicated that residing in an urban area increases the risk of childhood overweight and obesity in middle-income countries [22-24]. Moreover, this study also found that overweight clusters (Cluster 1) were mainly detected in districts and cities in West Sumatra Province, while obesity clusters were mainly found in North Sumatra Province (Tables 1-2). The study found that compared to children living outside these clusters, children within these two clusters had 1.4 and 1.5 times higher risk of overweight and obesity, respectively. In addition, the provinces of East Java, Central Java, West Java, and North Sumatra have become the provinces with the highest number of districts and cities included in the clusters of overweight and obesity. Policymakers should consider prioritizing intervention and prevention efforts in these regions to address overweight and obesity. Despite the growing visibility of the obesity epidemic in Indonesia, there has been limited political and financial support allocated to addressing this issue. The National and Medium-Term Development Plan (RPJMN) for 2020-2024 gave more attention to addressing obesity in the population aged 18 years and above rather than to children [25].

Moreover, national prevention programs aimed at tackling overweight and obesity, such as the National Movement to Reduce Obesity (GENTAS), encountered challenges in implementing appropriate interventions at the local level due to a lack of coordination between national and local authorities [26]. This is likely due to the large geographical size of Indonesia, which contains numerous islands that are separated from each other, making coordination and collaboration between different regions challenging. Thus, prioritizing the cluster areas identified in this study can be a highly valuable strategy for the government to improve coordination between national and local authorities and implement an effective intervention plan.

Previous studies [27,28] have outlined specific regions in Indonesia as targets for addressing childhood overweight and obesity primarily based on prevalence rates. A study revealed the most developed regions showed a notably greater incidence of child overweight and obesity compared with the least developed regions, by as much as 1.37-1.38 times [28]. Interestingly, our study found that the districts with the highest prevalences of overweight (Tolikara District) and obesity (Paniai District) were not included in the primary cluster. This is because the primary cluster was not solely determined by a higher disease rate; rather, the analysis also accounted for neighboring areas with an elevated risk, as detected by the restricted flexible scan statistics methods. Because phenomena are often observed in geographically proximate regions [29], it is crucial to account for obesity and overweight prevalences and neighboring areas when selecting regions for an overweight and obesity prevention program.

This study marks a pioneering effort in the context of Indonesia, as it utilizes geographical information to examine childhood obesity and overweight patterns. The clusters identified in this study could be designated as priority areas for the implementation of health promotion by public health nurses in Indonesia. Indonesian children are particularly in need of policies that promote healthy behavior due to the high prevalence of sedentary activity and unhealthy food consumption in Indonesia [3]. This study has several potential limitations. First, we did not incorporate any covariates to analyze clusters of overweight and obesity due to a lack of such open data at the same geographical unit. Hence, the results of this study did not consider other variables (e.g., age, gender, race/ethnicity, socioeconomic status, and population density) [12] that could potentially influence clusters of overweight and obesity. Future studies can use Welfare Statistics 2018 [14] data to include socioeconomic variables in the spatial cluster analysis of overweight and obesity at the provincial level in Indonesia. Second, this study explored the spatial clustering of overweight and obesity with large-scale administrative units like districts and cities. Future studies should consider examining these issues at an individual level, utilizing point data to gather more detailed insight at a smaller scale. In addition, our analysis was restricted to a single year of data due to availability; therefore, we were unable to validate the temporal and spatial trends related to overweight and obesity. Third, in Basic Health Research 2018, insufficient information was available regarding the equipment used to measure weight and height, as well as the specific procedure for measuring children (e.g., whether children were measured while dressed or undressed). This absence of detailed information may introduce potential bias, as different equipment and measurement methods can lead to varied results.

## Conclusions

In conclusion, this pioneering study provides valuable insights into methods of identifying clusters of childhood overweight and obesity prevalences. For this study, the primary concentrations were identified in Western Indonesia. Prioritizing interventions in the identified cluster areas offers a strategic approach to enhance coordination between national and local authorities. Comprehensive, region-specific interventions are essential to address the escalating issue of overweight and obesity in Indonesia.
